# Multimodal imaging findings in a case of severe Central Serous Chorioretinopathy in an uncomplicated pregnancy

**DOI:** 10.1186/s12886-015-0169-x

**Published:** 2015-12-22

**Authors:** Emilia Maggio, Antonio Polito, Maria C. Freno, Grazia Pertile

**Affiliations:** Sacrocuore Hospital, Via Don Sempreboni 5, Negrar, 37024 Verona Italy

**Keywords:** Central Serous Chorioretinopathy, Pregnancy, Subretinal exudates

## Abstract

**Background:**

Central Serous Chorioretinopathy (CSC) has been previously reported as an infrequent complication of pregnancy that usually resolves spontaneously after delivery, with minimal or no sequel. We report a case of a severe form of CSC in an uncomplicated pregnancy with extensive subfoveal exudates and severe permanent visual loss. Multimodal imaging techniques, including color and red-free photographs, near-infrared reflectance, fluorescein angiography, and spectral-domain optical coherence tomography, were performed and the findings were correlated to the changes in visual acuity.

**Case presentation:**

A 35-year-old pregnant woman presented with loss of vision and metamorphopsia in her left eye. Fundus examination showed subfoveal severe exudation with a posterior pole serous detachment. Optical coherence tomography (OCT) showed macular neurosensory detachment with central highly reflective sub-retinal material. Multimodal fundus pictures and angiograms revealed distinct clinical features of the disease during both the acute and final phase. The disease spontaneously resolved after delivery with regression of the subretinal fluid and the disappearance of subfoveal exudates. Nevertheless, because of severe atrophic macular changes and subfoveal fibrosis, no improvement of visual acuity was noted.

**Conclusion:**

Severe variants of CSC may also present in cases of uncomplicated pregnancy and result in a poor prognosis. Recognising these presentations of CSC is critical to avoid improper management. Multimodal imaging may help to clarify the diagnosis and highlight the clinical features.

## Background

Central serous chorioretinopathy (CSC) has been previously reported as an infrequent complication of pregnancy. However, it has been more commonly described as a benign, transient condition, which usually resolves spontaneously after delivery, with minimal or no sequel [1–5]. We report a case of a pregnant patient who developed a more severe form of CSC with extensive subfoveal exudates and severe permanent visual loss.

## Case presentation

**Fig. 1:**
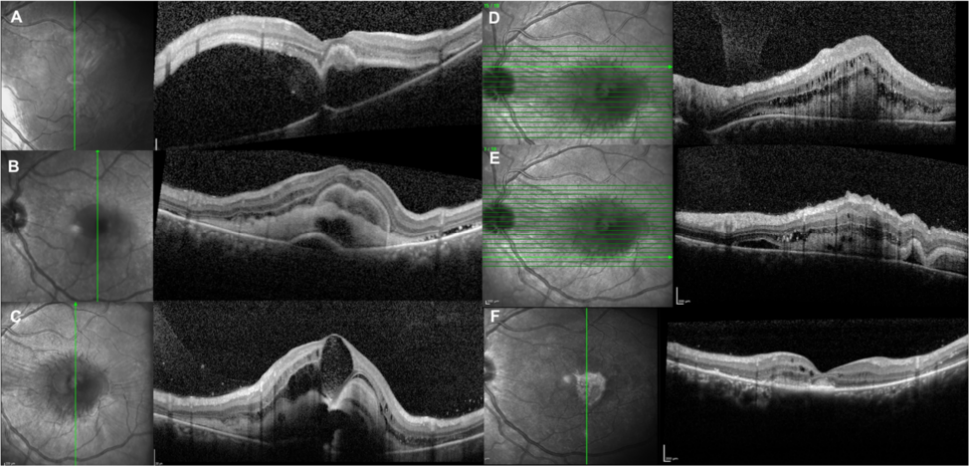
OCT findings in the acute and final phase of the disease. **a** Optical coherence tomography (OCT) at baseline showing a large serous elevation of the neurosensory retina in the macular area with central highly reflective sub-retinal material. BCVA: 20/200. **b** OCT at 1 week follow-up visit showing resolution of subretinal fluid and the appearance of double layered subretinal material, presumably due to an increase in fibrinous subretinal exudates. BCVA: 20/200. **c** OCT scan through the fovea at 1 month follow-up visit. Large foveal cystoid spaces associated with subfoveal exudates shaped like multiple concentric highly reflective layers masking deeper poorly defined reflective material on top of the retinal pigment epithelium. BCVA: 20/250. **d**-**e** OCT scans superior and inferior to the fovea at 1 month follow-up visit showing subretinal exudation and intraretinal cystoid spaces. **f** OCT after delivery showing complete resolution of subretinal fibrin, severely damaged outer retinal layers and subfoveal fibrosis. BCVA: 20/250

A 35-year-old pregnant woman was admitted to our clinic complaining of visual loss and metamorphopsia in her left eye of five days’ duration. Retinal detachment with macular hole had been diagnosed at another clinic. Her previous systemic and ocular history was unremarkable. On ophthalmic examination best corrected visual acuity (BCVA) was 20/20 in the right eye and 20/200 in the left eye with small refractive error. Examination of the anterior segment was unremarkable and there were no signs of intraocular inflammation in both eyes. Ophthalmoscopy revealed yellow-white round subfoveal exudation with a localised posterior serous detachment extending up to the inferior arcade in the left eye. Optical coherence tomography (OCT) showed serous elevation of the sensory retina in the macular area involving the fovea with central highly reflective sub-retinal material (Fig. 1a). Her right eye was normal.

**Fig. 2:**
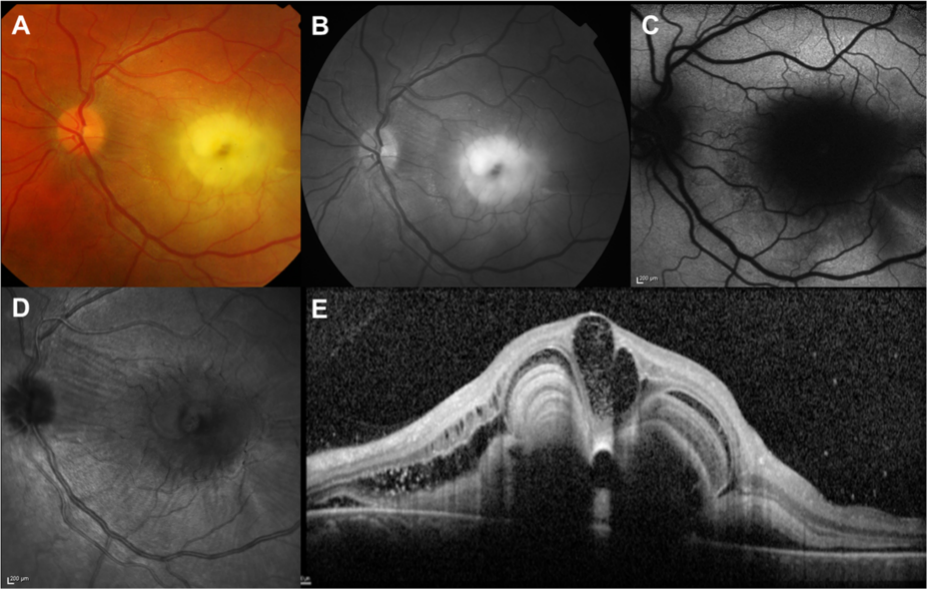
Multimodal imaging findings in the acute phase of the disease. **a** Color photograph showing yellow-white round subfoveal exudation. **b** Red free photograph improves the visualization of exudates. **c** Central hypoautofluorescence due to masking effect corresponding to the area of exudation. **d** An irregular, round dark area with low infrared reflectance centered on the fovea within normal fundus reflectance. **e** OCT scan showing intraretinal cystoid spaces and subfoveal exudates shaped in multiple concentric highly reflective layers

**Fig. 3:**
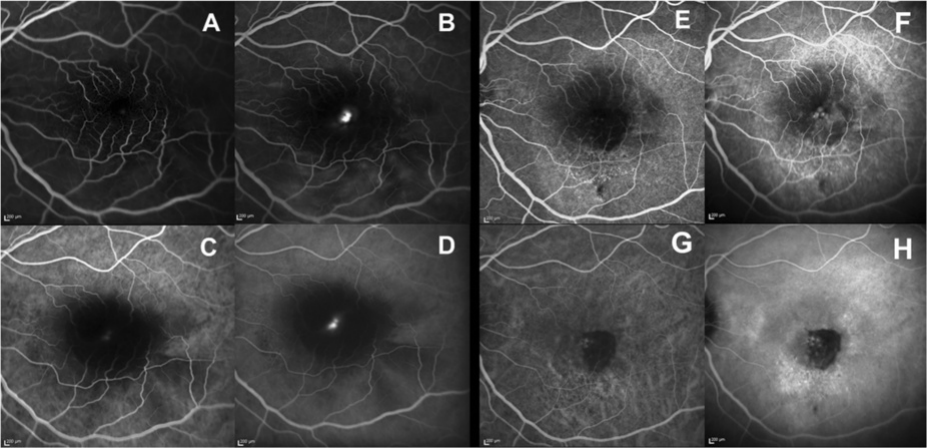
FAG-ICG findings in the acute and final phase of the disease. **a**-**d** FA (*top*) and ICG (*bottom*) in the acute phase of the disease showing a large central area of hypofluorescence due to the masking effect since the early angiograms (**a**, **c**) associated with pooling and mild leakage of dye at the foveal center in the late angiograms (**b**, **d**). **e**-**h** FA (*top*) and ICG (*bottom*) in the late phase of the disease showing central hypofluorescence, corresponding to the area of subretinal fibrosis, within a large hyperfluorescent area, associated with mild pooling in the late angiograms (early angiograms in **e**, **g** images; late angiograms in **f**, **h**)

Initially the patient refused fluorescein angiography (FA), despite being informed there was no risk to the fetus. One week later OCT showed resolution of subretinal fluid and an increase in hyper-reflective material (Fig. 1b). One month later, BCVA decreased to 20/250 and fibrinous exudates further increased, forming multiple dome-shaped-layered subretinal structures, masking further subretinal material. Moreover, large intraretinal cystoid spaces were detected at the foveal center on OCT (Fig. 1c-d-e). Color and red-free (RF) photographs, autofluorescence (AF) and near-infrared reflectance (NIR) images and OCT findings are shown in Fig. 2. FA and indocyanine green angiography (ICG) were performed and showed a large area of hypofluorescence due to the masking effect, associated with pooling and mild leakage of dye in the late phase of the angiogram (Fig. 3). The table provides a summary of the multimodal imaging findings during the acute and final phases of the disease (Table 1). Because of the pattern of leakage and the lumpy bumpy subretinal material on OCT, the possible presence of type 1 choroidal neovascularization (CNV) was discussed. However, given the absence of evidence of a vascular network on ICG, the absence of hard exudates or hemorrhages and the absence of irregular pigment epithelial detachments with hyper-reflective content compatible with the presence of neovascular tissue in any of the OCT volume scans, the diagnosis of CNV was then excluded.

**Table 1:** Abnormal findings of multimodal imaging in the acute and final phases of the disease

Diagnostic tool	Abnormal findings	
	Acute phase	Final phase
Color photograph	Yellow-white subfoveal exudation	Yellow-grey central subretinal fibrosis
AF	Central hypoautofluorescence due to masking effect	Central hypoautofluorence surrounded by a large hyperautofluorescent area
RF	Central light area corresponding to the exudates	Central dark round area corresponding to the fibrosis
NIR	Central dark area with low infrared reflectance	Central bright area with increased infrared reflectance
SD-OCT	Subretinal exudates shaped as multiple concentric highly reflective layers, intraretinal cystoid spaces, choroidal thickening	Subfoveal fibrosis, intraretinal cystoid spaces, severely damaged outer retinal layers
FA-ICG	Central hypofluorescence, pooling and mild leakage of dye, absence of a neovascular network	Central hypofluorescence, a surrounding large hyperfluorescent area, mild pooling

Systemic investigation did not reveal any abnormality. At her initial visit to our clinic the patient was in the fifth month of pregnancy. Routine blood tests were normal, as were immunological exams, including complement fractions C3 and C4, rheumatoid factor, IgG, IgM, antinuclear antibodies, ENA, antiphospholipid antibodies. Serology tests for herpes simplex, herpes zoster, cytomegalovirus, syphilis, rubella, Lyme disease, HIV, tubercolosis, toxoplasma were negative. The patient denied intake of any systemic or topical medication. Gynaecological evaluation did not show any evidence of obstetric complications.

**Fig. 4:**
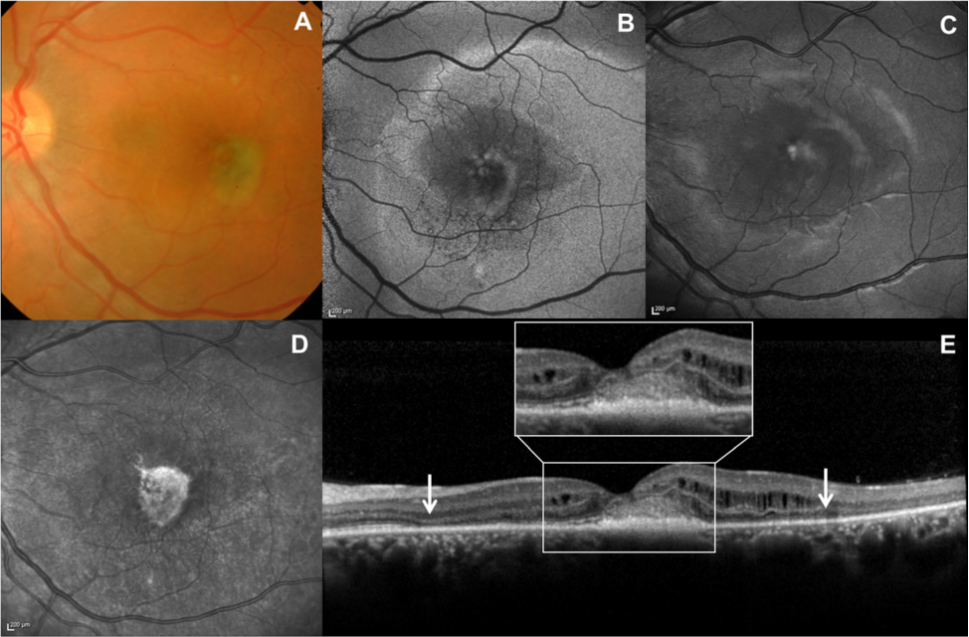
Multimodal imaging findings in the final phase of the disease. **a** Color photograph showing yellow-grey subretinal fibrosis. **b** Central hypoautofluorence corresponding to the subretinal fibrosis surrounded by a large hyperautofluorescent area corresponding to the outer retinal layers’ s atrophy. **c** Red free photograph showing the central area of subretinal fibrosis. **d** IR showing a central bright area with increased infrared reflectance. **e** OCT scan showing subfoveal fibrosis (magnified area), intraretinal cystoid spaces and severely damaged outer retinal layers (*white arrows*)

**Fig. 5:**
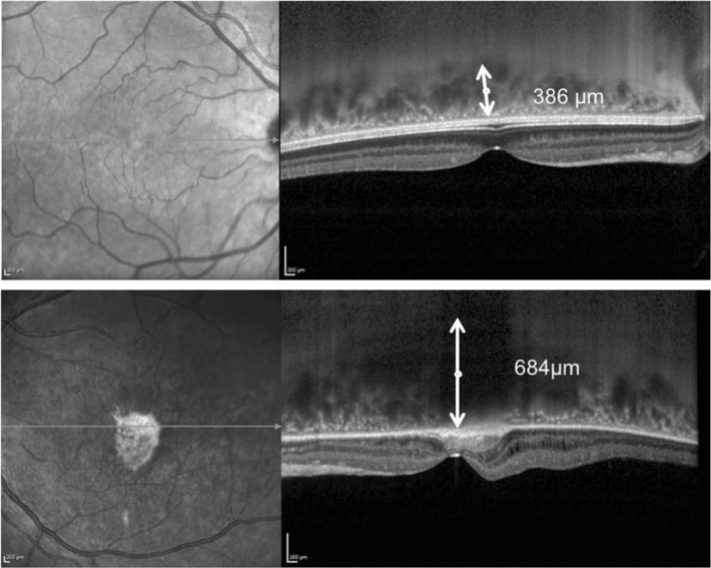
OCT after delivery. Inverted images acquired to measure the choroidal thickness show significant choroidal thickening in the left eye

The clinical features led us to a diagnosis of CSC in pregnancy. The pregnancy and the severity and extent of subretinal exudation ruled out therapy. The patient was evaluated by OCT monthly during pregnancy and quarterly postpartum. After delivery, CSC spontaneously resolved; however, despite the resolution of the subretinal fluid and the disappearance of subfoveal exudates, BCVA did not improve, due to the severe atrophic macular changes. Color photograph, RF, AF, NIR and OCT findings after delivery are shown in Fig. 4. OCT showed severely damaged outer retinal layers in the entire macular area, subfoveal fibrosis and persistent choroidal thickening in the left eye (Fig. 1f). The subfoveal choroidal thickness measured vertically from the outer border of the retinal pigment epithelium to the inner border of the sclera was 684 μm in the left eye, but 386 μm in the right eye (Fig. 5). As Enhanced Depth Imaging-OCT software was not commercially available at the time of the patient’s presentation, inverted images were acquired and used to measure choroidal thickness. FA and ICG were performed and showed central hypofluorescence corresponding to the area of subretinal fibrosis due to the masking effect, surrounded by a large hyperfluorescent area corresponding to the outer retinal layers’s atrophy; mild pooling was observed in the late angiograms (Fig. 3).

At the last FU, two years after delivery, visual acuity was stable in left eye with no recurrent episodes of CSC observed in this eye.

## Conclusions

Hormonal, hemodynamic, vascular and immunological changes occur during pregnancy that can be associated with ocular changes or with a worsening of pre-existing conditions [3]. CSC has been previously described as a possible ocular complication of pregnancy with an annual incidence of 0.008 % [4]. The disease usually resolves spontaneously after delivery, with minimal or no sequel. Quillen and Gass reported a small percentage (5 %) of pregnant patients with CSC who developed a more severe form with extensive retinal detachments and severe visual loss [5].

In the previous literature, the occurrence of sub-retinal (presumably fibrinous) exudates is also common in CSC accompanying pregnancy (50-90 %) - with or without a previous history of corticosteroid use [2, 5]. The nature of these exudates and the reason for their increased prevalence during pregnancy remain unknown, as are the risk factors for CSC development in pregnancy. As corticosteroids are a recognized risk factor for CSC, the cortisol levels increase throughout the third trimester and/or in the final trimester - possibly the result of increased catecholamines levels during pregnancy - may have a role in the pathogenesis of the disease in pregnant women. Moreover, a possible choroidal vessel dysfunction resulting from vasomotor stress in pregnancy might also be involved, since congestion of the choroidal vessels is thought to play a primary pathophysiologic role in this disorder. However, the complete disappearance of exudates and the resolution of subretinal fluid with minimal or no sequel has been commonly described in previous reports of CSC in pregnancy. We describe a more severe presentation of the disease, with extensive subretinal exudates and poor final visual outcome.

Exaggerated variants of CSC with bullous retinal detachments, subretinal fibrinous exudates and large, single or multiple pigment epithelial detachments, have been described rarely following corticosteroid therapy [6], organ transplantation [7] or haemodialysis [8]. Our patient had no history of corticosteroid use and her pregnancy had been uncomplicated.

Because of the atypical clinical findings, these variants of CSC may be misinterpreted as choroidal neovascularization, retinitis or retinal detachments. Multimodal imaging may help to clarify the diagnosis in cases of severe forms of CSC. Diverse imaging modalities may reveal distinct pathological details, making it easier to highlight the clinical features of the disease. Color and RF photographs, AF and NIR images clearly show the characteristics of the subfoveal exudates. FA and ICG help to exclude the presence of CNV by establishing the absence of a vascular network and the absence of hemorrhages. OCT volume scans may also help avoid misinterpretation, as they allow a better definition of the exudates and confirm the absence of CNV. EDI OCT (replaced by inverted images in our case) may improve the visualization of the choroidal anatomy and thus the identification of choroidal swelling, which is a common finding in CSC.

The present case shows that, although rare, severe variants of CSC may present in cases of uncomplicated pregnancy and result in a poor prognosis. Moreover, it highlights the importance of recognising atypical presentations of CSC in order to avoid misdiagnoses and improper management.

## Consent

Written informed consent was obtained from the patient for publication of this case report and any accompanying images. A copy of the written consent is available for review by the Editor of this journal.

## Abbreviations

BCVA: best corrected visual acuity
CNV: choroidal neovascularization
CSC: Central Serous Chorioretinopathy
FA: fluorescein angiography
ICG: indocyanine green angiography
OCT: optical coherence tomography
